# Teams in Transition: Increasing Role of Advanced Practice Providers in Antimicrobial Use and Infectious Diseases Consultation

**DOI:** 10.1093/ofid/ofae141

**Published:** 2024-04-04

**Authors:** Reinaldo Perez, Michael E Yarrington, Connor R Deri, Michael J Smith, Jillian Hayes, Rebekah H Wrenn, Rebekah W Moehring

**Affiliations:** Division of Infectious Diseases, Department of Medicine, Duke University Medical Center, Durham, North Carolina, USA; Department of Medicine, Duke Center for Antimicrobial Stewardship and Infection Prevention, Department of Medicine, Durham, North Carolina, USA; Division of Infectious Diseases, Department of Medicine, Duke University Medical Center, Durham, North Carolina, USA; Department of Medicine, Duke Center for Antimicrobial Stewardship and Infection Prevention, Department of Medicine, Durham, North Carolina, USA; Department of Pharmacy, Duke University Medical Center, Durham, North Carolina, USA; Department of Medicine, Duke Center for Antimicrobial Stewardship and Infection Prevention, Department of Medicine, Durham, North Carolina, USA; Division of Pediatric Infectious Diseases, Duke University Medical Center, Durham, North Carolina, USA; Department of Pharmacy, Duke University Medical Center, Durham, North Carolina, USA; Department of Pharmacy, Duke University Medical Center, Durham, North Carolina, USA; Division of Infectious Diseases, Department of Medicine, Duke University Medical Center, Durham, North Carolina, USA; Department of Medicine, Duke Center for Antimicrobial Stewardship and Infection Prevention, Department of Medicine, Durham, North Carolina, USA

**Keywords:** advanced practice provider, antimicrobial stewardship, antimicrobial days of therapy, ID consultaiton, interdisciplinary teams

## Abstract

**Background:**

Advanced practice providers (APPs) have taken on increasing responsibilities as primary team members in acute care hospitals, but the impact of this practice shift on antimicrobial prescribing and infectious diseases (ID) consultation requests is unknown. Here we describe longitudinal trends in antimicrobial days of therapy (DOT) and ID consultation by attributed provider type in 3 hospitals.

**Methods:**

We performed a retrospective time series analysis of antimicrobial use and ID consultation from July 2015 to June 2022 at a major university hospital and 2 community hospitals. We evaluated antimicrobial DOT and ID consultation over time and assessed attribution to 3 groups of providers: attending physicians, trainees, and APPs. We used multinomial logistic regression to measure changes in percentage of DOT and ID consultation across the clinician groups over time using physicians as the referent.

**Results:**

Baseline distribution of antimicrobial DOT and ID consultation varied by practice setting, but all subgroups showed increases in the proportion attributable to APPs. Large increases were seen in the rate of ID consultation, increasing by >30% during the study period. At our university hospital, by study end >40% of new ID consults and restricted antimicrobial days were attributed to APPs.

**Conclusions:**

Hospitals had differing baseline patterns of DOT attributed to provider groups, but all experienced increases in DOT attributed to APPs. Similar increases were seen in changes to ID consultation. APPs have increasing involvement in antimicrobial use decisions in the inpatient setting and should be engaged in future antimicrobial stewardship initiatives.

Advanced practice providers (APPs), including nurse practitioners and physician assistants, have taken on increasing responsibilities in the management of hospitalized inpatients over the last decade. The Society of Hospital Medicine reported that >80% of hospital medicine groups utilize APPs and >40% of physician assistants identify the hospital as their primary clinical setting [1]. A number of studies of APP-based hospital care teams demonstrated equivalent medical outcomes plus decreased length of stay and higher rates of discharge to home as compared with physician solo practice [2–5]. Given anticipated physician shortages and hospital financial incentives, employing multidisciplinary teams with APPs is likely to further rise [1, 6]. Little is known, however, about the impact of this clinical provider workforce change on the outcomes of inpatient antimicrobial use and infectious diseases (ID) consultation requests.

Previous research has shown that workplace culture, hierarchy, communication behaviors, and provider perceptions affect the antimicrobial decision-making process [7, 8]. These factors may vary by the type of clinician and one’s training background. For example, one survey reported that when compared with physicians, APPs were less likely to (1) report feeling that their training in antimicrobial prescribing was adequate, (2) feel that antibiotics were overused, and (3) correctly choose empiric therapy based on an antibiogram [9]. Additionally, outpatient data suggest a difference in appropriateness of antibiotic prescribing based on provider type, though such comparisons are not available from inpatient settings [10, 11]. Understanding the composition of decision makers that hospital-based antimicrobial stewardship programs and ID consultants aim to support has significant implications for antimicrobial stewardship program strategy, development, and implementation.

We sought to better understand the changing roles of provider groups in antimicrobial use and ID consult requests across our 3-hospital health system. Our main aim was to describe the longitudinal trends in antimicrobial days and ID consultation stratified by provider type: attending physician, APP, and trainee. We hypothesized that we would see an increase in antimicrobial use and ID consultation originating from APP providers over the study period.

## METHODS

### Design and Setting

We performed a retrospective descriptive time series analysis of a 7-year period from July 2015 to June 2022. The analysis included our entire health care system, consisting of a 1000-bed academic medical center, a 350-bed community hospital with significant trainee presence, and a 180-bed community hospital with minimal trainee presence. We included data from all inpatient admissions for the study period. Inpatient APPs in our health system function under the supervision of physicians, but the team structure and degree of oversight vary significantly by service line. They generally serve as the “first call” clinician on subspecialty wards, general medicine wards, and critical care units with responsibilities similar to physician trainees. The majority work in surgical and subspecialty areas, with a small proportion working on hospitalist services.

Antimicrobial days of therapy (DOT) were defined per methods of the National Healthcare Safety Network (NHSN) [12]. We looked at 4 classes of antimicrobials: antibacterials, antifungals, antivirals, and “protected agents.” Antibacterial and antifungal agent groups were defined by NHSN lists, while the antiviral group included anti-influenza and antiherpesvirus agents in addition to agents in the preexisting NHSN category. We defined protected agents as those targeted by hospital antimicrobial stewardship program policy (eg, requiring preauthorization). ID consultation rates were based on new ID consult orders placed into the electronic medical record. Provider type was defined by electronic health record user profiles and placed into 3 categories: physician, trainee (residents, fellows, medical students), and APP (nurse practitioners, physician assistants, and nurse anesthetists).

### Analysis

For our antimicrobial use outcomes, we first evaluated DOT/1000 days present by agent group over time to assess quarterly rate trends for all 3 hospitals in aggregate. Then, for each hospital, we calculated the percentage of total DOT by provider type for each agent group and evaluated the relative distribution. We used multinomial logistic regression to measure changes across clinician groups over time, using physicians as the referent. We used a similar approach for ID consultations, starting with an overall assessment of consult rate trends, followed by an analysis of provider distribution trends at each hospital. All computations were completed in SAS (SAS 9.4, SAS Institute). Our primary outcome was the change in percentage of antimicrobial DOT or ID consults attributable to APPs, with physicians as the referent. This analysis was done for each of the 3 hospitals and each of the antimicrobial therapeutic classes. The study protocol was deemed exempt by the Duke University Institutional Review Board.

## RESULTS

### Antimicrobial Use

During our study period, the overall antimicrobial use across the health system varied greatly by agent group. Antibacterial DOT/1000 days present decreased by an average of 0.7% per quarter for a 17% decrease total (rate ratio, 0.83; 95% CI, .80–.86). The protected agent group showed similar changes with a 0.7% quarterly decrease and a 17% total decrease (rate ratio, 0.83; 95% CI, .75–.92). In contrast, antiviral DOT/1000 days present rose by an average of 1.2% per quarter for an overall increase of 38% (rate ratio, 1.38; 95% CI, 1.12–1.69), and antifungal use remained relatively stable (rate ratio, 1.03; 95% CI, .96–1.11).

**Figure 1.**
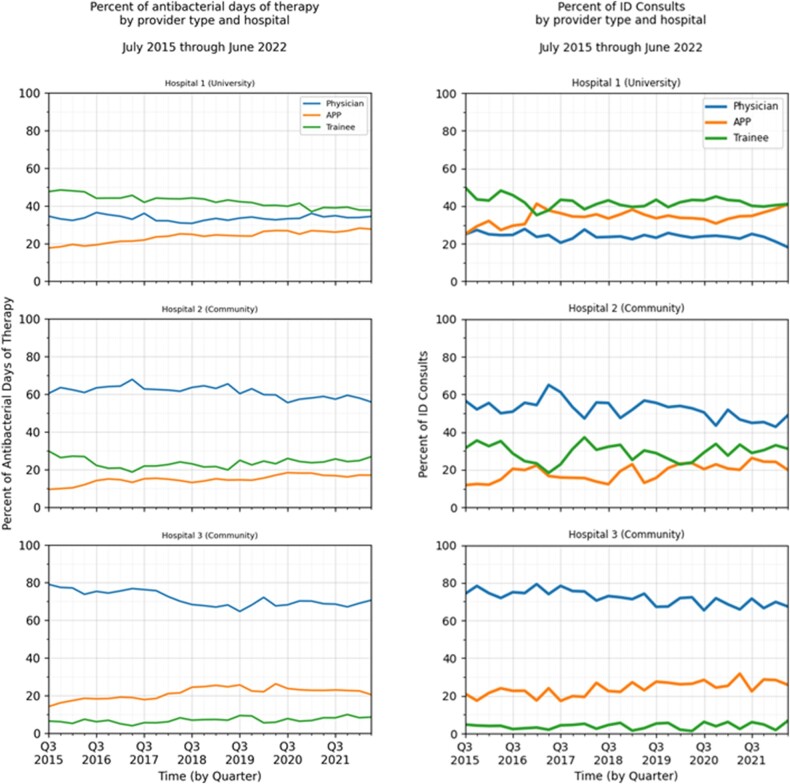
Shifts in provider type attribution of antimicrobial days of therapy and infectious diseases (ID) consults at 3 hospitals. APP, advanced practice provider.

The initial distribution of antimicrobial DOT by provider type depended on the hospital practice model. At the university hospital, antimicrobial ordering largely depended on APPs and trainees, while community hospitals were more dependent on physicians. Despite this, all 3 hospitals saw shifts in their antimicrobial DOT toward APPs for each antimicrobial group (Table 1). Our regression model demonstrated that the shifts from physician to APP were statistically significant, with the odds ratio for antibacterial ordering moving to APPs by an average of 1.5% to 2% per quarter and for ordering of protected agents moving to APPs at 3% to 4% per quarter (Table 2). The proportion of ordering attributable to trainees was highly variable by hospital and agent group. The shift to APPs was most dramatic among protected agents and at the university hospital, with 41% of protected agent DOT being ordered by APPs by the end of the study period. Sample depictions of these shifts over time are available in Figure 1.

**Table 1.** DOT by Provider Type Across 3 Hospitals and 28 Quarters: July 2015–June 2022

	DOT (%), Quarter 1			DOT (%), Quarter 28			
Antimicrobial: Hospital	Physician	Trainee	APP	Physician	Trainee	APP	Change for APPs, %^a^
Antibacterials							
1: University	18 008 (35)	24 813 (48)	9236 (18)	17 661 (34)	19 363 (38)	14 216 (28)	+10
2: Community	6184 (61)	3058 (30)	978 (10)	5559 (56)	2675 (27)	1699 (17)	+7
3: Community	6627 (79)	549 (7)	1195 (14)	7020 (71)	860 (9)	2046 (21)	+7
Antifungals							
1: University	2373 (35)	3296 (49)	1630 (24)	1875 (22)	2504 (29)	2919 (35)	+11
2: Community	203 (61)	114 (34)	14 (4)	190 (60)	62 (20)	60 (19)	+15
3: Community	324 (74)	61 (14)	52 (12)	367 (67)	48 (9)	130 (24)	+12
Antivirals							
1: University	2788 (39)	2709 (38)	1636 (23)	2707 (26)	3558 (35)	3969 (39)	+16
2: Community	112 (60)	63 (34)	10 (5)	269 (75)	70 (19)	20 (5)	0
3: Community	171 (85)	3 (2)	26 (13)	524 (82)	21 (3)	95 (15)	+2
Protected							
1: University	4450 (38)	4681 (39)	2719 (23)	2758 (26)	3600 (33)	4434 (41)	+15
2: Community	272 (56)	174 (36)	42 (9)	432 (66)	113 (17)	112 (17)	+8
3: Community	396 (79)	58 (11)	46 (9)	448 (80)	17 (3)	93 (17)	+8

Abbreviations: APP, advanced practice provider; DOT, days of therapy.

^a^Change in percentage of DOT for APPs: quarters 1 vs 28.

**Table 2.** Change in Percentage of DOT by Hospital and Provider Type

	Quarterly Change in Percentage of DOT, Odds Ratio (95% CI)	
Antimicrobial: Hospital	Trainee vs Physician	APP vs Physician
Antibacterials		
1: University	0.991 (.991–.992)	1.015 (1.014–1.015)
2: Community	1.005 (1.004–1.006)	1.020 (1.019–1.021)
3: Community	1.020 (1.018–1.022)	1.017 (1.016–1.019)
Antifungals		
1: University	1.014 (1.013–1.016)	1.033 (1.032–1.034)
2: Community	0.993 (.987–.998)	1.030 (1.023–1.038)
3: Community	1.016 (1.008–1.023)	1.029 (1.023–1.034)
Antivirals		
1: University	1.018 (1.017–1.020)	1.039 (1.038–1.041)
2: Community	0.980 (.974–.986)	1.016 (1.008–1.025)
3: Community	1.049 (1.034–1.065)	1.009 (1.003–1.015)
Protected		
1: University	1.018 (1.017–1.019)	1.038 (1.037–1.039)
2: Community	0.994 (.990–.998)	1.027 (1.021–1.033)
3: Community	1.018 (1.008–1.028)	1.042 (1.036–1.048)

In the multinomial logistic regression model, physician was the referent category among the provider groups.

Abbreviations: APP, advanced practice provider; DOT, days of therapy.

### ID Consultation

Over the 7 years of our study, the rate of new ID consults/1000 patient days present rose at all sites, but the degree varied by hospital. The university hospital experienced an average quarterly increase in consultation rate of 1.9%, for an overall increase of 66.9% (rate ratio, 1.66; 95% CI, 1.55–1.78). Our community hospital with a significant trainee presence (hospital 2) similarly saw a quarterly increase of 2.1%, for a total increase of 77.1% (rate ratio, 1.77; 95% CI, 1.55–2.01). In contrast, our community hospital without a trainee presence (hospital 3) showed only a 0.8% quarterly increase in consultation rate, for an overall increase of 22.7% (rate ratio, 1.22; 95% CI, 1.07–1.39). Across the health system, this averaged to a 35% increase in the ID consultation rate over the 7-year study period.

Similar to the antimicrobial use data, baseline proportions of ID consult ordering varied per hospital dependent on staffing models. Despite this, all 3 facilities saw increases in the proportions of ID consults ordered by APPs, with an average quarterly shift toward APPs of 1% to 2% relative to physicians. While the proportional change was most dramatic at hospital 2, the absolute shift was most dramatic at our university hospital, with 41% of ID consults being ordered by APPs (Tables 3 and 4). Sample depictions of these shifts over time are available in Figure 1.

**Table 3.** ID Consults by Provider Type Across 3 Hospitals and 28 Quarters: July 2015–June 2022

	ID Consults (%), Quarter 1			ID Consults (%), Quarter 28			
Hospital	Physician	Trainee	APP	Physician	Trainee	APP	Change for APPs, %^a^
1: University	254 (25)	509 (50)	255 (25)	299 (18)	674 (41)	671 (41)	+16
2: Community	96 (57)	53 (31)	20 (12)	144 (49)	92 (31)	59 (20)	+8
3: Community	140 (74)	9 (5)	40 (21)	172 (67)	17 (7)	66 (26)	+5

Abbreviations: APP, advanced practice provider; ID, infectious diseases.

^a^Change in percentage of ID consults for APPs: quarters 1 vs 28.

**Table 4.** Change in Percentage of ID Consults by Hospital and Provider Type: July 2015–June 2022

	Quarterly Change in Percentage of ID Consults, Odds Ratio (95% CI)	
Hospital	Trainee vs Physician	APP vs Physician
1: University	1.003 (1.000–1.006)	1.011 (1.008–1.015)
2: Community	1.009 (1.002–1.016)	1.028 (1.019–1.037)
3: Community	1.015 (.999–1.030)	1.018 (1.011–1.025)

In the multinomial logistic regression model, physician was the referent category among the provider groups.

Abbreviations: APP, advanced practice provider; ID, infectious diseases.

## DISCUSSION

The proportion of antimicrobial use attributable to APPs substantially rose over the study period across all 3 hospitals in our health system and for each of our defined antimicrobial subgroups. The rate of ID consultation also increased substantially, and a significantly rising percentage of those consults were requested by APPs. This study is one of the first to describe the magnitude of inpatient provider type shifts over a relatively short period and its relation to antimicrobial use. Our findings emphasize the growing roles of inpatient APPs, showcasing their importance in supporting stewardship goals, designing stewardship interventions, and achieving process improvements.

Existing data on antibiotic use do not report on shifts in the antimicrobial prescriber workforce or ID consultation requesters over time. A limited number of prior studies have compared physician and APP antimicrobial prescribing rates, though results are mixed. Most data come from the outpatient setting and describe the relative appropriateness of antibiotics for treating upper respiratory tract infections. Two studies in adult populations at major East Coast centers showed that APPs prescribed antibiotics at a rate about 15% higher than their physician colleagues [10, 11]. In contrast, a study of pediatric patients based on Kentucky Medicaid data showed that physicians were 25% more likely than their nurse practitioner colleagues to prescribe an antibiotic for upper respiratory tract infection diagnoses [13]. While we identified no studies comparing the appropriateness of inpatient antimicrobial prescribing, a recent survey of inpatient APPs at 5 acute care hospitals suggested that differences exist. Only 37% of APPs surveyed felt that their training in antimicrobial prescribing was adequate, with 62% able to correctly choose empiric therapy based on an antibiogram [9]. While our study was unable to clearly delineate attribution of the antimicrobial decision making or the appropriateness of use, the findings emphasize the importance of investigating these questions further as APPs take on larger responsibility in the inpatient setting.

We report the expanding role of APPs in antimicrobial prescribing and ID consultation requests in the setting of an overall 17% decrease in antibacterial and protected agent use and a 35% rise in ID consultation rates across the study period. Many other temporally related activities occurred during the 7-year period, including active stewardship programs in all 3 hospitals and the COVID-19 pandemic. The observed trends, while suggestive, do not offer direct evidence of an association among the increasing inpatient role of APPs, rising rates of ID consultation, or decreasing use of antimicrobials. However, these data demonstrate a need to further evaluate and adjust our antimicrobial stewardship program strategy to better meet the needs of our changing provider population. We believe that there are several opportunities to engage with APPs to further stewardship principles and goals. Previous studies have shown that inclusion of APPs into trauma and orthopedic service lines led to decreased rates of urinary tract infection, increased rates of appropriate perioperative antimicrobial prophylaxis, and a higher rate of prophylaxis for deep vein thrombosis [14, 15]. In addition to other data on quality metrics, this could suggest, for example, that APPs may be more receptive to process-oriented strategies such as checklists, order sets, or guidelines [2]. Stewardship teams should consider how APPs are being captured or excluded by current communication strategies and how the needs of this population may be different from physicians or pharmacists; they should also examine their role in supporting antibiotic decisions and determine if different strategies may be more effective in interfacing with APPs. To this end, we believe that more qualitative study could better identify if drivers of antibiotic decisions are different for APPs as opposed to other provider types.

The rise in ID consultation rate has implications beyond stewardship team strategy and is relevant to all ID clinicians. Previous studies that examined overall resource utilization of APP-based teams typically found no difference in cost or evidence of cost savings; however, few specifically analyzed subspecialty consultation [5, 16]. One recent study compared hospitalist, resident, and APP general medicine services at a single institution. At this hospital, APP teams had a 15% higher consultation rate than hospitalist teams [17]. While we were unable to make this estimate directly, our health system hospitals observed a significant longitudinal increase in ID consultation rate at the same time as a significant increase in the percentage of consults requested by APPs. More evidence is needed to determine if APP-based care teams result in higher consultation rates. Yet, ID divisions and hospital systems should consider the expanding role of APPs when accounting for consult team staffing. Furthermore, qualitative research may shed light on best practices when consulting with and supporting APPs as compared with physicians.

For our own antimicrobial stewardship program, we take these data as a call to action to improve our processes and enhance APP inclusion. APPs staff the bulk of complex subspecialty services at our institution (eg, oncology services and solid organ transplant services), partially explaining their large role in ordering of protected antimicrobials. Our current outreach efforts have consisted of a multifaceted approach, including partnering with institutional APP leadership for improved communication, conducting focus groups with APP-based teams to assess their familiarity with stewardship resources, and identifying local champions on specific service lines to offer feedback when new interventions are proposed. We have also proposed inclusion of an APP representative as a standing member of our pharmacy and therapeutics committee to ensure that APPs’ perspective is represented. Other efforts at engagement include surveying APPs to better understand the unique dynamics of different service lines. Our efforts are only beginning, but clearly more opportunity to engage and support our APP partners exists.

Our study has a number of limitations. First, we were unable to obtain specific patient day denominators for solo physician, resident, and APP teams and were thus unable to compare rates or appropriateness of antimicrobial or consult ordering by provider type. Second, attribution of consult request or antimicrobial use was based on the initial ordering provider in the electronic medical record. This may not accurately reflect the clinician driving the decision to use antimicrobials or order an ID consult, as team handoffs are frequent and many decisions are team based. Finally, this study occurred in a single university health system and may not be representative of changes occurring across the US health care system. Despite these limitations, these data were deeply informative for our own practice setting, and national trends of inpatient APP utilization suggest that these findings could be similar and relevant to peer institutions.

APPs serve a critical role in addressing our country's physician shortage and have rapidly stepped into a diverse array of inpatient roles. In our health system, they have come to represent a substantial percentage of our antimicrobial ordering and frequently serve as our primary contacts for inpatient ID consults. Preliminary research suggests that there may be differences in the ordering practices of APPs and in their comfort with antimicrobial prescribing in general. Further understanding of these differences will be essential to the success of our interdisciplinary teams of the future.

## Notes

***Patient consent statement.*** The study was evaluated by the Duke University Institutional Review Board and deemed exempt with a waiver of consent, given that no personal health information was utilized.
